# Animal-assisted therapy with farm animals for persons with psychiatric disorders: effects on self-efficacy, coping ability and quality of life, a randomized controlled trial

**DOI:** 10.1186/1745-0179-4-9

**Published:** 2008-04-11

**Authors:** Bente Berget, Øivind Ekeberg, Bjarne O Braastad

**Affiliations:** 1 Norwegian University of Life Sciences, Department of Animal and Aquacultural Sciences, P.O. Box 5003, NO-1432 Ås, Norway; 2 University of Oslo, Department of Behavioural Sciences in Medicine, P.O. Box 1111 Blindern, NO-0317, Oslo, Norway

## Abstract

**Background:**

The benefits of Animal-Assisted Therapy (AAT) for humans with mental disorders have been well-documented using cats and dogs, but there is a complete lack of controlled studies using farm animals as therapeutic agents for psychiatric patients. The study was developed in the context of Green care, a concept that involves the use of farm animals, plants, gardens, or the landscape in recreational or work-related interventions for different target groups of clients in cooperation with health authorities. The present study aimed at examining effects of a 12-week intervention with farm animals on self-efficacy, coping ability and quality of life among adult psychiatric patients with a variety of psychiatric diagnoses.

**Methods:**

The study was a randomized controlled trial and follow-up. Ninety patients (59 women and 31 men) with schizophrenia, affective disorders, anxiety, and personality disorders completed questionnaires to assess self-efficacy (Generalized Self-Efficacy Scale; GSE), coping ability (Coping Strategies Scale), and quality of life (Quality of Life Scale; QOLS-N) before, at the end of intervention, and at six months follow-up. Two-thirds of the patients (N = 60) were given interventions; the remaining served as controls.

**Results:**

There was significant increase in self-efficacy in the treatment group but not in the control group from before intervention (SB) to six months follow-up (SSMA), (SSMA-SB; F_1,55 _= 4.20, p= 0.05) and from end of intervention (SA) to follow-up (SSMA-SA; F_1,55 _= 5.6, p= 0.02). There was significant increase in coping ability within the treatment group between before intervention and follow-up (SSMA-SB = 2.7, t = 2.31, p = 0.03), whereas no changes in quality of life was found. There were no significant changes in any of the variables during the intervention.

**Conclusion:**

AAT with farm animals may have positive influences on self-efficacy and coping ability among psychiatric patients with long lasting psychiatric symptoms.

## Background

The utilization of agricultural farms as a basis for promoting human mental and physical health in cooperation with health authorities is growing in several countries in Europe and in the United States of America. In some countries this is called *Green care*, a concept which is not restricted to the use of animals, but also includes plants, gardens, forests, and the landscape. Historically, Green care farms were associated with hospitals, psychiatric departments and other health institutions. Today, most Green care projects involve community gardens, city farms, allotment gardens and farms. Because many Green care farms are rather small compared with traditional farms, there is often a diversity of activities, with the possibility of meaningful work for different people and target groups. Other positive experiences with Green care like self-esteem, responsibility and sense of purpose are similar in the different countries [1]. During the last decade, an increasing number of persons with mental disorders work with farm animals as part of their therapy [2-5].

Although Animal-Assisted Therapy (AAT) for humans with mental disorders has been well documented with cats and dogs, there is a complete lack of controlled studies of farm animals as therapeutic agents for psychiatric patients. Previous studies of AAT with companion animals have documented that human-animal interaction may decrease stress levels [6-12], and is shown to improve self-confidence, social competence and quality of life [13,14]. As it is shown that different types of animals may have different impact on people's health [15,16], it is therefore worth investigating to what extent contact and work with farm animals will contribute to self-efficacy, coping ability and quality of life among psychiatric patients.

In AAT with farm animals, we suggest that the combined effect of both contact and work with the animals can affect the patients positively; by providing a source of physical contact with a living "other", and increased coping ability and self-esteem through routines that include feeding, milking, and caring for other living creatures. Green care programs with farm animals can be important supplements to a traditional psychiatric treatment in reaching the goals of self-esteem and coping ability. An intervention with farm animals will shift from care in an institutional regime to increased social integration and normalisation of care.

We have earlier reported increased intensity and exactness of work by patients with psychiatric disorders in a 12-week intervention with farm animals [17,18]. The patients also showed significantly lower anxiety at the end of the intervention and at follow-up six months after the end of the intervention compared with baseline. No such changes were found for the control group.

In the present paper based on the same sample we report on effects on self-efficacy, coping ability and quality of life of a 12-week intervention with farm animals for adult psychiatric patients. The aims of the present study were as follows:

1) To examine whether animal-assisted therapy for psychiatric patients was associated with higher self-efficacy, coping ability and quality of life after treatment and at six months follow-up.

2) To assess if there were different treatment responses in the different diagnostic groups.

3) To investigate the relationship between changes in self-efficacy, coping ability and quality of life and specific questions related to the intervention.

For checklist of the randomized trial, see Additional file 1.

## Methods

### Sample

Therapists recruited the candidate patients, and informed written consent was obtained. The Norwegian Data Inspectorate and the Regional Committee for Medical Research Ethics approved the project, and the study took part between February 1, 2003 and January 1, 2006. Ninety adult psychiatric patients with a variety of psychiatric diagnoses were requited, and there were 59 (65.6%) women and 31 (34.4%) men [Additional file 2]. The mean age (± SD) was 34.7 ± 10.7 (range 18–58) years. In the patient group there were 14 inpatients (15.5 %) and 76 outpatients (84.5 %). Diagnoses were made by the treating psychiatrists using the ICD 10 criteria [19], and the main diagnoses were 34 (37.7 %) schizophrenia and schizotypal disorders (F 20–29), 22 (24.4 %) affective disorders (F 30–39), 10 (11.1 %) anxiety and stress-related disorders (F 40–49), and 22 (24.4 %) disorders of adult personality and behaviour (F 60–69). There was one patient with eating disorders (F 50) in the treatment group who dropped out, and one patient with behavioural disorders due to psychoactive substance use (F11) in the control group who completed the project. This patient was omitted when analysing for effects of diagnosis. For 15 randomly chosen patients, the diagnoses were checked for consensus between treating and research psychiatrists using the ICD 10 criteria [19] and all were found to accord.

More than 50 % of the patients had been ill for more than five years, and 72 % had been treated in psychiatric health institutions for more than three years. As much as 83% of the 90 included patients received daily medication, mainly antipsychotics (53 %), antidepressants (50 %), sedatives (35 %) and mood stabilizers (27 %). Exclusion criteria were: (a) age less than 18 years, (b) acute psychotic disorders, (c) mental retardation, (d) serious drug addiction, and (e) being in a job during the six last months prior to start of intervention. No minimal levels of symptoms were required.

### Randomization

The patients were randomized by computerized random numbers by BOB to intervention with farm animals or to a control group. There was two-thirds (60 patients) to AAT and one-third (30 patients) to control. The reason for this division was that we expected more drop-outs in the treatment than in the control group due to experience in a pilot project. Diagnoses and other demographic variables were unknown during the randomization process. The patients were well informed of the possibilities of getting into either of the two groups, and no one withdrew after the results of the randomization.

### The farms and farmers

Among the 15 recruited farmers there were seven women and eight men. Only two farmers had earlier experience with psychiatric patients. The main productions were dairy cows (N = 10 farms, mean 20 animals), specialised meat production with cattle (N = 2, mean 22 animals), sheep (N = 2, mean 30 animals), or horses (N = 1, mean 18 animals). All farmers had small animals like rabbits, poultry, pigs, cats or dogs as a part of the milieu on the farm.

### Intervention

The treatment group received standard therapy (individual, group therapy or other kinds of therapy) and stable medical treatment in addition to the intervention. The control group got treatment as usual. No significant differences in standard therapy, medication, educational level and outpatient/inpatient ratio at baseline were found between the groups.

The patients visited a farm for three hours twice a week for 12 weeks to work with the farm animals. One or two patients visited the farm at each time. The patients were only working with the animals; they were not allowed to do other kinds of farm work. The farmers were told that the work should depend on the patient's coping ability and interest, and that patients should have the opportunity of physical contact with the animals. The farmers were always close to the patients during the work with the animals to ensure that there were no risks related to the contact with the animals. The patients were also trained in the working routines during the first week of the intervention. An overview over the most frequent behaviours in the interaction with the animals is given in Table 1.

**Table 1:** Different behaviours that were observed during the interactions with the animals

*Behaviour*	Explanation
1. Physical contact with the animals	Patting, brushing, washing, looking after=?, nursing, or saddling or riding horses.
2. Communication	Verbalization, visual contact.
3. Moving the animals	Behaviours that include moving animals between different places in the cowshed, and between different pastures.
4. Feeding	Feeding adult animals with concentrate or forage, or milk feeding the small animals.
5. Go/stand/run or sit down	The participants moved around in the cowshed to bring tools and straw to clean the boxes, or remained inactive.
6. Cleaning	Cleaning the cowshed or washing buckets and bottles.
7. Milking	All routines connected to the milking procedure.
8. Receiving instructions	Receiving instructions from the farmer.
9. Various	Behaviours that occur rarely, like filming the animals or taking pictures of the animals.
10. Threatening behaviour directed from the animals.	Receiving threatening or aggressive behaviour or signals from the animals.

### Outcome measures

Three different inventories were used. These instruments are all tested for their validity and reliability and are commonly used in psychiatric research and clinical practice. The patients' scores were obtained before the intervention (SB), in the end of the intervention (after: SA), and six months after the end of the intervention (SSMA). Self-efficacy was measured with the Generalized Self-Efficacy Scale (GSE) assessing the strength of an individual's belief in his/her ability to respond to novel or difficult situations [20]. The scale comprises ten items, and the patient responds to a 4-point scale from 1 'not at all true' to 4' exactly true'. The score range is 10–40. Coping was measured using the Coping Strategies Scale of the Pressure Management Indicator [21,22]. The scale comprises six items measuring control coping and four items measuring support coping and the patient responds to a 6-point scale from 1 'never used by me' to 6 'very much used by me'. The score range is 10–60. A Norwegian version of Quality of Life Scale (QOLS-N) was used comprising 16 items and reflecting relations to other humans, work, and leisure [23]. The patient responds on a 7-point scale with 1 'very content' to 7 'very discontent' with the score range of 16–112. High scores reflect high degree of self-efficacy, coping and quality of life. Thirty of the patients completing the intervention answered a final questionnaire related to their experience with the intervention. The following questions were used: "To what extent has the contact and work with the animals affected your coping ability in daily life; – your mood; – your self-esteem; – your working ability; – made you more extrovert and talkative?" and "To what extent has the physical contact with the animals been important to you as part of the work?". The patients answered on a 5-point scale from 1 'much worse/very little' to 5 'much better/very much', with point 3 'no change'.

### Statistics

Analysis of variance (ANOVA) was performed by standard least square means with three difference scores (intervention period: SA-SB; intervention + post-intervention: SSMA-SB, and post-intervention: SSMA-SA) as the dependent variables, patient group (treatment: T/control: C) and diagnosis as the independent variables, and the interaction between patient group and diagnosis. Matched-paired t-tests were performed to examine differences in means between time points for each group (T or C). Spearman correlation analyses were used to measure correlations between self-efficacy and coping, and to relate the difference in the patient's scores in the intervention period (SA-SB) to their answers on the experience of the intervention (treatment group only). Chi-square tests analysed for differences between completers and dropouts. The level of significance was set at p < 0.05. All analyses were conducted with SAS [24].

## Results

There were 41 completers (68 %) in the treatment group and 28 (93 %) in the control group. The scores of the completers on self-efficacy (GSE), coping strategy (Coping Strategies Scale), and quality of life (QOLS-N) at different times are presented in Table 2.

**Table 2:** Scores in Self-efficacy (GSE), Quality of Life Scale (QOLS-N) and Coping Strategies Scale before the intervention, at the end of the intervention, and six months after the end of the intervention for the treatment (N = 41) and control (N = 28) groups (mean ± SD).^1^

Variables	Score before (SB)	Score after (SA)	D.F	F (SA-SB)	P	Score six months after end of intervention (SSMA)	D.F	F (SSMA-SB)	P	D.F.	F (SSMA-SA)	P
**Self-efficacy (GSE)**												
Treatment group	23.1 ± 5.12	23.5 ± 6.56	1,60	0.02	n.s.	25.7 ± 5.93	1,55	4.20	0.05	1,55	5.6	0.02
Control group	25.6 ± 6.40	25.3 ± 6.62				25.4 ± 5.92						

**Quality of Life Scale (QOLS-N)**												
Treatment group	64.3 ± 14.93	64.3 ± 17.09	1,60	0.49	n.s.	66.7 ± 16.86	1,57	0.38	n.s	1,57	0.38	n.s
Control group	63.2 ± 14.06	64.4 ± 13.52				66.0 ± 15.25						

**Coping Strategies Scale**												
Treatment group	31.6 ± 8.51	32.8 ± 8.67	1,60	0.01	n.s.	34.3 ± 8.10	1,57	0.79	n.s.	1,57	0.39	n.s
Control group	32.2 ± 7.38	31.4 ± 8.69				31.6 ± 8.02						

^1 ^Analysis of variance (ANOVA) is used to test the differences in means between registration times and groups.

There were no significant baseline differences between the groups in any of the inventories.

For the GSE scores, the ANOVA analysis revealed no effect of treatment during intervention (SA-SB) compared with the control group (Table 2). However, the difference in scores between six months after intervention and before (SSMA-SB) was significantly higher in the treatment than in the control group, reflecting a larger increase in self-efficacy for the treatment group. A similar effect was found in the post-treatment period (SSMA-SA). Within the treatment group there was no significant increase in self-efficacy during the intervention. At six months follow-up the GSE scores were significantly higher than the baseline (SSMA-SB = 2.6, t = 3.68, p = 0.001) and higher than at the end of intervention (SSMA-SA = 2.2, t = 4.38, p = 0.0001). For the control group there were no significant differences between any of the time points.

For the F30-group the ANOVA analysis of GSE scores showed significance during the intervention (T_F30(SA-SB) _(24.8–22.6) = 2.2, C_F30(SA-SB) _(25.4–27.3) = -1.9, F = 5.01, p = 0.03), and from before to six months after (T_F30(SSMA-SB) _(28.3–22.6) = 5.7, C_F30(SSMA-SB) _(27.3–27.3) = 0.0, F = 6.36, p = 0.01), reflecting an increased self-efficacy for the affective patients in the treatment group. Within this patient group, GSE showed nearly significant increase in the intervention period for the treatment group (SA-SB = 24.8–22.6 = 2.2, t = 2.09, p = 0.066), and significant increases between before and six months after (SSMA-SB = 28.3–22.6 = 5.7, t = 3.56, p = 0.006) and during the post-treatment period (SSMA-SA = 28.3–24.8 = 3.5, t = 2.54, p = 0.03). For the control group no such change was found. There was no significant change in scores for any of the other diagnoses.

For the Quality of Life Scale (QOLS-N) there was no significant difference in scores between or within the total treatment or control groups for any periods. However, the ANOVA analysis showed significant difference in the F30-group between follow-up and before intervention (T_F30(SSMA-SB)_(71.9–58.5) = 13.4, C_F30(SSMA-SB) _(64.6–67.4) = -2.8, F = 6.30, p = 0.01), reflecting an increase in QOLS-N for the affective patients in the treatment group.

For the Coping Strategies Scale the ANOVA analysis revealed no treatment effect for any of the periods (Table 2). However, there was significant positive change in scores for the treatment group between before and six months after (SSMA-SB = 2.7, t = 2.31, p = 0.03). For the control group there were no significant differences between any of the registration times. There were no significant changes in scores in any of the different diagnoses.

Patients with the largest increase in self-efficacy during the intervention also showed the largest increase in coping (r_s _= 0.45, p = 0.0029). A similar effect was also found between baseline and six months after (SSMA-SB, r_s _= 0.57, p = 0.0002), and in the post-treatment period (SSMA-SA, r_s _= 0.64, p < 0.0001). There were no such correlations for the controls in any of the registration periods. There were also significant correlations between the difference in GSE scores during the intervention (SA-SB) and the patients' answers related to the intervention (treatment group only). Patients with the largest increase in GSE reported the largest increase in coping ability in daily life (r_s _= 0.40, p = 0.03). Similarly, the patients with the largest increase in coping strategy reported the largest improvement in mood (r_s _= 0.42, p = 0.02), and they favoured to a larger extent physical contact with the animals (r_s _= 0.40, p = 0.03).

The dropouts in the treatment and control group left the project after a range of 1–6 weeks, and there were 31(75.6 %) women and 10 (24.4 %) men among the completers in the treatment group, and 17 (60.7 %) women and 11 (39.3 %) men in the control group. The main reasons for dropping out of the intervention was little interest in the animal species on the farm (26.7 % answering 'very much the reason'), that the work was boring (20 % answering 'very much'), and private reasons (26.7 % answering 'very much'). Comparison between completers and the drop-outs showed significant differences between the groups in institutional connection, manifested as higher drop-out among the hospitalised patients (χ^2 ^= 13.01 p = 0.006). There was also a higher degree of patients using sleeping medicine among the dropouts (χ^2 ^= 3.77 p = 0.05). There were no other differences in medication.

## Discussion

During the six months follow-up period self-efficacy was significantly better in the treatment group, but not in the control group. According to Bandura, self-efficacy refers to the expectation that one can effectively cope with and master situations through one's own personal efforts [25,26]. The finding that self-efficacy was higher during the six months follow-up period in the treatment but not in the control group, could be attributed to several explanations. One is that the patients may have learned new tasks during the intervention, and afterwards felt more self-confident. Another potential explanation is effects of the ordinary psychiatric treatment being improved by the AAT intervention, i.e. the AAT serving as a catalyst for positive development in the patient. A third explanation is that the contact with the animals may have produced a pleasurably experienced social interaction that made the patients less afraid of new situations, and that the effects first appeared during the follow-up period. A similar pattern was also found for coping strategy within the treatment group in the period between before the intervention and six months follow-up. A study of Ventura *et al. *[27] concluded that psychotic patients who had greater feelings of self-efficacy and problem-focused coping strategies, appeared to be more likely to cope with day-to-day stressors. Our study also showed that the patients in the treatment group with the largest increase in self-efficacy during both the intervention and the follow-up period, showed the largest increase in coping strategy in the same periods, whereas such effects were not found for the controls. Similarly the patients with the largest increase in GSE scores also reported the largest increase in coping ability in daily life when relating the questions to the intervention experience. Likewise, the patients with the largest increase in coping also reported favouring physical contact with the animals. Although there was no significance during intervention, these findings indicate that AAT with farm animals offer a combined effect of both contact and work with the animals that have positive influences on the development of self-efficacy and coping ability.

There was no effect on quality of life at any of the registration periods for the total patient group. This is in contrast to earlier controlled studies with pets [28,29]. We found however, as for GSE, increased quality of life among persons with affective disorders. These findings indicate that patients in this diagnostic category profit most on the treatment. This is in accordance with the study of Antonioli and Revely [30] who found significant reduction in depression during AAT with dolphins compared with the control group.

It is a question whether the length of the intervention in our study was too short, or the frequency of farm visits too low to get significant differences between the groups during the intervention. The delayed effect in GSE and Coping Strategy Scale could indicate this, but this very effect also indicated that the intervention with farm animals did have some effects additional to the usual treatments.

Even if the results on self-efficacy and coping are rather moderate, they are positive based on the limited sample size and the rather unspecific intervention. According to Wilson and Barker [31], questions must be raised whether the characteristics of the animals, the farmer, the settings and the interaction among these variables may influence the assessed outcomes. In addition, most of the patients (72 %) had been treated in psychiatric health institutions for more than three years, which makes it unlikely to achieve a rapid and great improvement. We found a significantly higher drop-out rate among the hospitalised patients compared with the outpatients. This indicates that some inpatients were in an unstable phase. In future studies these might have to be excluded to minimize the drop-out rate.

The strengths of this study are that it is the first randomized controlled follow-up design with farm animals, and that the logistics have contributed to increased cooperation between health institutions, therapists and farmers, and made Green care more accepted among the health professions in Norway. Further strengths were that the health outcome measures were based on validated standardized instruments, the completeness of the assessments, and the relatively moderate drop-out rate. Limitations were the moderate number of patients in the different diagnostic categories, and the inherent inability to blind the active treatment.

## Conclusion

The results of this study suggest that AAT with farm animals may be a useful addition to traditional psychiatric treatment, particularly for patients with affective disorders. Self-efficacy was higher at follow-up compared with baseline and at the end of the intervention in the treatment group but not in the control group. The patients with the highest increase in self-efficacy during intervention reported the largest increase in coping ability. Further controlled studies are needed for confirmation and to more accurately define the treatment parameters and the psychiatric population with the greatest potential of benefiting from an intervention with farm animals.

## Competing interests

The author(s) declare that they have no competing interests.

## Authors' contributions

BB has undertaken the conception and design of the study, the data collection and analysis. She has also drafted the manuscript. ØE and BB have contributed to the design of the study and to the drafting and revision of the manuscript. All authors read and approved the final version.

## Supplementary Material

Additional file 1 CONSORT Statement 2001 – Checklist. Checklist describing where in the article important aspects of the randomized controlled trial are found. Click here for file

Additional file 2 The Consort E-Flowchart Aug. 2005. Flowchart of the research design. Click here for file
