# Supplementary material for: Animal-assisted therapy with farm animals for persons with psychiatric disorders: effects on self-efficacy, coping ability and quality of life, a randomized controlled trial
Source: Clin Pract Epidemiol Ment Health. 2008 Apr 11;4:9. doi: 10.1186/1745-0179-4-9 (PMC2323374; doi:10.1186/1745-0179-4-9)
Supplement: Additional file 2 — The Consort E-Flowchart Aug. 2005. Flowchart of the research design. [file 1745-0179-4-9-S2.doc]

**The Consort E-Flowchart Aug. 2005**

Assessed for eligibility (n= )

Excluded (n= )

Not meeting inclusion criteria

(n= )

Refused to participate

(n= )

Other reasons

(n= )

**Allocation**

**Analysis**

**Follow-Up**

**Enrollment**

Analyzed (n=41 )

Excluded from analysis (n= 0 )

Give reasons

Lost to follow-up (n= 0 )

Give reasons

Discontinued intervention

(n= 19 )

# Give reasons: bored or didn’t appreciate the animals

Allocated to intervention

(n= 60 )

Received allocated intervention

(n= 60 )

Did not receive allocated intervention

(n= 0 )

Give reasons

Lost to follow-up (n= 0 )

Give reasons

Discontinued intervention

(n= 2 )

Give reasons

Allocated to control

(n= 30 )

Received allocated to control

(n= 30 )

Did not receive allocated to control

(n= 0 )

Give reasons

Analyzed (n= 28 )

Excluded from analysis (n= 0 )

Give reasons

Is it Randomized?
